# An Approach to In Vitro Manufacturing of Hypertrophic Cartilage Matrix for Bone Repair

**DOI:** 10.3390/bioengineering4020035

**Published:** 2017-04-20

**Authors:** Bach Quang Le, Clemens van Blitterswijk, Jan de Boer

**Affiliations:** 1 Department of Tissue Regeneration, MIRA Institute for Biomedical Technology and Technical Medicine, University of Twente, Postbus 217, 7500 AE Enschede, The Netherlands; c.vanblitterswijk@maastrichtuniversity.nl; 2 Department of Complex Tissue Regeneration, MERLN Institute, University of Maastricht, P.O. Box 616, 6200 MD Maastricht, The Netherlands; 3 Laboratory for Cell Biology-inspired Tissue Engineering, MERLN Institute, University of Maastricht, P.O. Box 616, 6200 MD Maastricht, The Netherlands

**Keywords:** Hypertrophic cartilage, ATDC5, decellularized matrix, devitalized matrix, tissue engineering, bone regeneration

## Abstract

Devitalized hypertrophic cartilage matrix (DCM) is an attractive concept for an off-the-shelf bone graft substitute. Upon implantation, DCM can trigger the natural endochondral ossification process, but only when the hypertrophic cartilage matrix has been reconstituted correctly. In vivo hypertrophic differentiation has been reported for multiple cell types but up-scaling and in vivo devitalization remain a big challenge. To this end, we developed a micro tissue-engineered cartilage (MiTEC) model using the chondrogenic cell line ATDC5. Micro-aggregates of ATDC5 cells (approximately 1000 cells per aggregate) were cultured on a 3% agarose mold consisting of 1585 microwells, each measuring 400 µm in diameter. Chondrogenic differentiation was strongly enhanced using media supplemented with combinations of growth factors e.g., insulin, transforming growth factor beta and dexamethasone. Next, mineralization was induced by supplying the culture medium with beta-glycerophosphate, and finally we boosted the secretion of proangiogenic growth factors using the hypoxia mimetic phenanthroline in the final stage of in vivo culture. Then, ATDC5 aggregates were devitalized by freeze/thawing or sodium dodecyl sulfate treatment before co-culturing with human mesenchymal stromal cells (hMSCs). We observed a strong effect on chondrogenic differentiation of hMSCs. Using this MiTEC model, we were able to not only upscale the production of cartilage to a clinically relevant amount but were also able to vary the cartilage matrix composition in different ways, making MiTEC an ideal model to develop DCM as a bone graft substitute.

## 1. Introduction

Endochondral ossification is a fascinating phenomenon in which a cartilage template is remodeled into bone tissue by a highly regulated mechanism. During embryonic development, endochondral ossification occurs in parallel to intramembranous ossification to build the skeletal system. In fracture healing, the process very much recapitulates embryonic development and thus usually results in complete restoration of the skeletal organ [1,2,3]. However, when bone defects reach critical sizes or when systemic factors such as osteoporosis presents, clinical intervention is necessary to augment bone healing [4]. Current options to treat bone defects range from autologous bone to synthetic osteoinductive materials with/without growth factors e.g., bone morphogenetic proteins [5]. Until now, there is no clinical treatment that utilizes endochondral ossification, specifically from a hypertrophic cartilage graft.

Previously, we have demonstrated the feasibility of tissue-engineering hypertrophic cartilage from mouse embryonic stem cells (mESCs) in vivo which then remodeled into new bone in vivo [6]. To this end, mESCs were chondrogenically differentiated for 2 to 3 weeks, followed by subcutaneous implantation in immunodeficient mice for 4 weeks. A high correlation was observed between the amount of hypertrophic cartilage created in vivo and the amount of bone detected in vivo. Similarly, Scotti et al. subcutaneously implanted bone marrow-derived human mesenchymal stromal cells (hMSCs) at various stages of chondrogenic differentiation [7]. They observed bone trabeculae only when the hMSCs differentiated in vivo into hypertrophic cartilage-like tissue. Weiss et al. differentiated ATDC5 pellets into hypertrophic cartilage in vivo before implantation in nude mice for 8 weeks [8]. They found mineralized tissue with active osteoclast resorption and neo-angiogenesis throughout the implants. These experiments and others [9,10,11,12] show proof of concept that in vivo cultured hypertrophic cartilage can continue endochondral ossification in vivo.

Live hypertrophic cartilage will form bone in vivo, but it has long been realized that even non-living tissues still contain bioactive components that can induce ossification. The most relevant example is demineralized bone matrix (DBM) developed by Urist in 1960. DBM is allogenic or xenogeneic bone processed to remove immunogenicity and preserve the osteoinductive bone morphogenetic proteins [13]. The chemistry of the remaining extracellular matrix (ECM), not the living cells, is sufficient to induce bone formation upon implantation and DBM is widely used as a bone graft substitute. Interestingly, hypertrophic cartilage tissue which is devitalized under certain conditions can also form bone in vivo. Bridge and Pritchard showed that epiphyseal plate or fracture callus devitalized with alcohol, acetone, HCl or heated to 55 °C consistently formed bone when implanted subcutaneously in the ear of rabbits [14]. Urist also observed bone formation when devitalized fracture callus was implanted into the anterior chamber of the eye of rats [15,16,17]. More recently, Bourgine et al. proved that hMSC-derived hypertrophic cartilage which was devitalized by inducible apoptosis could efficiently remodel to form de novo bone tissue of host origin, including mature vasculature and a hematopoietic compartment [18]. Cunniffe et al. created a porous scaffold by freeze-drying SDS-decellularized hMSC-engineered hypertrophic cartilage tissue. The scaffold induced vascularization and de novo mineral accumulation in a mouse ectopic model and formed full bridging (4 out of 8 animals) in a rat critically-sized femoral defect model [19]. Taken all together, these experiments demonstrated the feasibility of an osteoinductive material made of devitalized hypertrophic cartilage matrix (DCM), either harvested from biological sources or cultured in the lab.

A problem that will eventually hinder the utilization of DCM in the clinic is the ability to manufacture a great amount of this material in a reproducible and cost-effective manner. Making DCM from allografts or xenografts will not yield sufficient amounts since hypertrophic cartilage only presents in small amounts at the ends of long bones before adulthood. Thus, producing DCM from hypertrophic cartilage cultured in vivo seems to be a plausible answer. For this matter, choosing a cell type is the most critical step. One could start with undifferentiated stem cells, for examples embryonic (ES) cells or hMSCs, then induce chondrogenic differentiation until the cells reach the hypertrophic stage. The disadvantages of stem cells are, apart from the cost of maintaining stemness during storage and expansion, chondrogenic differentiation of ES cells is often heterogeneous [20] and donor variation in hMSCs is inevitable [21]. Starting with mature cells such as chondrocytes poses another problem; the cells have limited proliferation capacity before becoming senescent. While the choice of cell types remains to be elucidated, it is worth mentioning that the ideal cell type should have the following properties: (1) already committed to the chondrogenic lineage and (2) have unlimited capacity to divide. In the current study, although we do not claim to have the ideal cell type, we chose a cancer cell line—ATDC5—which has both mentioned properties to demonstrate our hypertrophic cartilage in vivo culturing model. The cell line ATDC5, derived from a mouse teratocarcinoma, is an excellent model to study skeletal development and has been utilized in over 200 studies to date [22]. ATDC5 has an intrinsic property to sequentially undergo hypertrophic chondrogenic differentiation [22,23,24]. Previously, we developed a spheroid culture system which allows us to assemble 3D, free-standing tissues with an intermediate complexity between 2D cell cultures and model organisms [25]. The advantages of this system and its application to date has been reviewed by Fennema et al. [26]. Here, we cultured ATDC5 in aggregates of approximately 1000–10,000 cells, which we named Micro Tissue-Engineered Cartilage (MiTEC). Compared to 2D culture models, MiTEC offers better cell-cell contacts and chondrogenic differentiation. Compared to traditional micro-mass or pellet models (of about 100,000 to 1,000,000 cells), MiTEC allows better diffusion of nutrients and growth factors. This model does not require additional scaffolding material but can be easily upscaled to produce a large amount of tissue mass applicable for all clinical purposes. Our long-term goal is to create an osteoinductive material made of devitalized ATDC5-derived hypertrophic cartilage that offers the ideal micro-environment for en route endochondral ossification. To reach this goal, in this study we optimized the in vivo production of MiTEC qualitatively and quantitatively. Medium composition and culture time was optimized to maximize hypertrophy; endogenous growth factor (VEGF) secretion was enhanced by treatment with hypoxia mimic molecules; and mineralization was induced with beta-glycerophosphate. MiTEC were devitalized either by freeze/thawing or by sodium dodecyl sulfate (SDS) treatment. Upon co-culturing with hMSCs, the SDS treated MiTEC had a strong effect on hMSC chondrogenic differentiation. Thus, we demonstrated the usability of the MiTEC model in optimizing and upscaling the in vivo culture of hypertrophic cartilage.

## 2. Materials and Methods

### 2.1. Cell Culture

ATDC5 cells (RIKEN cell bank, Ibaraki, Japan) were maintained in basic medium (BM) consisting of DMEM/F-12 GlutaMAX, 5% v/v fetal bovine serum (FBS, Lonza, Breda, Netherlands), 0.2 mM L-ascorbic acid-2 phosphate (ASAP), 100 U/mL penicillin and 100 µg/mL streptomycin (PS). Cells were grown at 37 °C in a humid atmosphere with 5% CO_2_. The medium was refreshed 2–3 times per week and the cells were used for further subculturing or cryopreservation upon reaching near confluence.

3% agarose (Life Technologies, Bleiswijk, Netherlands) chips were produced by replica molding from elastomeric stamps of poly(dimethylsiloxane) (PDMS; Sylgard 184, Dow Corning, Seneffe, Belgium); the stamps were replicated from either etched silicon wafers or SU-8/silicon wafers [25]. An agarose chip contains 1585 micro-wells, each measuring 400 µm in diameter and fitting in a well of a 12-well plate. To make aggregates, 1.5 million cells were seeded per agarose chip and centrifuged for 1 min at 300 G to collect the cells at the bottom of the micro-wells. This seeding density created an aggregate size of about 1,000 cells per aggregate, which was optimized for our 400 µm diameter micro-wells. The medium was changed every 1–2 days by pipetting from the side of the agarose chips without disturbing the aggregates. To optimize medium composition, 4 different formulations were used: (1) basic medium (BM); (2) BM supplemented with 1× insulin-transferrin-selenium (ITS) solution (IM); (3) BM supplemented with 0.1 µM dexamethasone (Dex) and 10 ng/mL transforming growth factor beta 3 (TGFβ3, R&D systems, 243-B3-010) (BC); and BM supplemented with 1× ITS, 0.1 µM Dex and 10 ng/mL TGFβ3 (IC).

Primary human mesenchymal stem cells (hMSCs) were obtained and isolated as described previously [27]. The use of bone marrow aspirates was approved for this whole study by the Medical Ethics Committee of Medisch Spectrum Twente and written informed consent was obtained from all patients. hMSCs were expanded at an initial seeding density of 1000 cells/cm^2^ in proliferation medium consisting of α-MEM, 10% v/v FBS (Lonza), 2 mM L-glutamine, 0.2 mM ASAP, PS, and 1 ng/mL recombinant human basic fibroblast growth factor (AbD Serotec, Kidlington, UK). For pellet mix-culture of hMSCs with devitalized or decellularized ATDC5 aggregates, a cell suspension of 2.5 × 10^5^ hMSCs (passage 2–5) was mixed with aggregates taken from 1 agarose chip in a 10-mL tube (Greiner, Kremsmünster, Austria) and was centrifuged for 3 min at 300 G to form a pellet. As a control, 2.5 × 10^5^ hMSCs alone were used to form a pellet in the same way. The chondrogenic medium for pellet co-culture consisted of DMEM high glucose, 100 µg/mL sodium pyruvate, 0.2 mM ASAP, PS, 1× ITS, 0.1 µM Dex, and 10 ng/mL TGFβ3 [28]. The basic medium for pellet co-culture was chondrogenic medium without Dex and TGFβ3. The pellets were cultured for 4 weeks and the medium was changed 2–3 times per week. All media and medium supplements were purchased from Life Technologies; all chemicals were purchased from Sigma-Aldrich (Zwijndrecht, Netherland), unless otherwise stated.

### 2.2. Devitalization and Decellularization

ATDC5 aggregates were cultured for 2 weeks in IC medium before devitalization by liquid nitrogen freeze/thawing or decellularization by sodium dodecyl sulfate (SDS). For devitalization, aggregates were collected and washed once with PBS followed by submersion in liquid nitrogen for 30 s and then in a 45°C water bath for 30 s, which was repeated 10 times. Decellularization was based on a protocol described by Kheir et al. [29]. Briefly, aggregates were subjected to 2 cycles of dry freeze/thaw followed by another 2 cycles of freeze/thaw in hypotonic buffer consisting of 10 mM Tris-HCl (pH 8.0) supplemented with 1× halt proteinase inhibitor cocktail (Thermo Scientific). Samples were frozen at −20 °C until crystal formation and were then thawed on the bench for 4 h. Then, aggregates were incubated in hypotonic buffer, 45 °C for 24 h, followed by 0.1% w/v SDS in hypotonic buffer at 45 °C for 24 h, with agitation. After washing with PBS twice for 30 min twice and incubation for 24 h at 45 °C, the aggregates were treated with a nuclease solution consisting of 50 mM Tris (pH 7.5), 10 mM magnesium chloride, 50 μg/mL bovine serum albumin, DNase (Sigma-Aldrich, 50 U/mL) and RNase (Sigma-Aldrich, 1U/mL), for 3 h at 37 °C. Finally, the aggregates were washed in PBS twice for 30 min and incubated for 24 h at 45 °C. All chemicals were purchased from Sigma-Aldrich unless otherwise stated.

### 2.3. Histological Staining and Immunostaining

Samples were fixed in 4% paraformaldehyde (Merck) in PBS. To avoid loss during histology processing, aggregates were incorporated in 0.5% agarose. Molten agarose (0.5% in ddH_2_O) was poured into 10 mL tubes containing aggregates followed by a quick centrifugation to collect all the aggregates at the bottom of the tubes. After solidification, the agarose portions holding all the aggregates were cut off for processing. Samples were dehydrated using sequential ethanol series, embedded in paraffin and 7 µm sections were cut using a microtome. Cartilage formation was visualized by 1% Alcian Blue staining in 3% acetic acid and 0.1% Nuclear Fast Red in 5% aluminum sulfate, which stained sulfated glycosaminoglycans blue and cell nuclei red. Mineralization was visualized by 2% Alizarin Red S, which stained calcium deposits orange-red. All chemicals were purchased from Sigma-Aldrich unless otherwise stated.

For immunostaining, the VECTASTAIN® Elite ABC-Peroxidase kit (Vector laboratories, Burlingame, CA, USA) was employed following the manufacturer’s protocol with some modifications. Briefly, antigen retrieval was performed by incubating sections with 0.1% w/v hyaluronidase (Sigma, H3506) and 0.1% w/v protease (Sigma, P5147) in PBS for 30 min at 37 °C. Sections were washed with 0.1% Tween-PBS and blocked with 0.3% H_2_O_2_-PBS for 10 min followed by 5% BSA-PBS for 30 min both at room temperature. Sections were incubated with primary antibody anti-collagen type 2 (1:100; Abcam; ab34712) or anti-collagen type 10 antibody (1:100; Abcam; ab58632), overnight at 4 °C in a humidified chamber. After washing, sections were incubated with biotinylated secondary antibody (1:100) followed by VECTASTAIN® ABC Reagent for 30 min each at RT. Brown staining was developed by incubating sections with peroxidase substrate solution for 5 min. Sections were rinsed with tap water and counterstained with haematoxylin (Sigma). Histological sections were analyzed by light microscopy (E600 Nikon).

### 2.4. Mineralization, Calcium Assay and Hydroxyproline Assay

Aggregates were cultured in IC medium for 2 or 3 weeks before switching to mineralization medium consisting of α-MEM, 5% v/v FBS, PS, 1× ITS, and 10 mM β-glycerophosphate. After 1 week culture in mineralization medium, the aggregates were collected for histological analysis, hydroxyproline assay and calcium assay. Samples were transferred to pressure-tight, teflon capped vials and hydrolyzed with 100 µL of 12 M HCl for 3 h at 120 °C. Five µL of the lysate was used for calcium analysis using the QuantiChrom Calcium Assay Kit (DICA-500) according to the manufacturer’s protocol. Briefly, free calcium specifically forms a stable blue colored complex with the phenolsulphonephthalein dye in the kit. The color intensity, measured at 612 nm, is directly proportional to the calcium concentration in the sample. A standard curve was generated using the Ca^2+^ standard. For hydroxyproline analysis, the Hydroxyproline Assay Kit (Biovision-K555) was used. Briefly, 10 µL of the hydrolyzed sample lysate was transferred to a 96-well plate and evaporated to dryness under vacuum. Next, chloramine T and 4-(dimethylamino)benzaldehyde (DMAB) reagent was added to each sample and incubated for 90 min at 60 °C. The reaction resulted in a colorimetric (560 nm) product, proportional to the hydroxyproline present. A standard curve was generated using 4-hydroxyproline as indicated in the manufacturer’s protocol.

### 2.5. Gene Expression Analysis

Cell aggregates or pellets were collected and washed once with PBS and then homogenized in Trizol reagent (Life Technologies) by crushing with a pestle and mortar under liquid nitrogen. After adding 20% v/v chloroform and centrifugation for 15 min at 11,000 g at 4 °C, the aqueous phase containing RNA was transferred to a new Eppendorf tube, combined with an equal volume of 70% ethanol, and then was loaded onto the RNA binding column of the RNA II nucleospin RNA isolation kit (Machery Nagel). The rest of the RNA isolation followed the kit protocol. RNA concentrations were measured using a ND100 spectrophotometer (Nanodrop1000). cDNA was synthesized from 1 µg of RNA, using iScript (BioRad) according to the manufacturer’s protocol. qPCR was performed using 50 ng of cDNA, 0.4 µM of each forward and reverse primer (Sigma Genosys), and 1× SensiMix SYBR&Fluorescein master mix (Bioline). Primer sequences are shown in Table 1. Real-time qPCR was performed in a Biorad My IQ5 machine (Biorad). Data was analyzed using the fit point method of the My IQ5 software. The baseline was calculated automatically by the software at the lower log-linear part above baseline noise and the crossing temperature (Ct value) was determined. Ct values were normalized to the Beta-2 microglobulin (B2M) housekeeping gene and ΔCt (Ct, control—Ct, sample) was used to calculate the up-regulation in gene expression.

### 2.6. ELISA

ATDC5 aggregates were cultured for 2 or 3 weeks in different media (BM, IM, BC or IC). Medium was changed every 2 days, with only half of the volume (1 mL) changed to avoid loss of the aggregates. VEGF was allowed to accumulate in medium for 2 days before the collection point. To boost VEGF secretion, ATDC5 aggregates were cultured in IC medium for 2 or 3 weeks, then the medium was supplemented with 50 µM phenanthroline (1,10-Phenanthroline monohydrate, Sigma-Aldrich) [30] and VEGF was allowed to accumulate for 2 days, 3 days or 6 days (with 1 medium change after 3 days) before the collection point. 1 mL of medium was collected from each chip and 50µL was assayed in duplicate using a mouse VEGF quantikine ELISA kit (R&D, MMV00) following the kit protocol.

### 2.7. Statistics

One-way or two-way ANOVA with Bonferroni post-test were performed using GraphPad Prism version 6.02 for Windows, GraphPad Software, La Jolla California USA, www.graphpad.com.

## 3. Results

### 3.1. Bulk Production of Micro-Tissue Engineered Cartilage (MiTEC)

**Figure 1:**
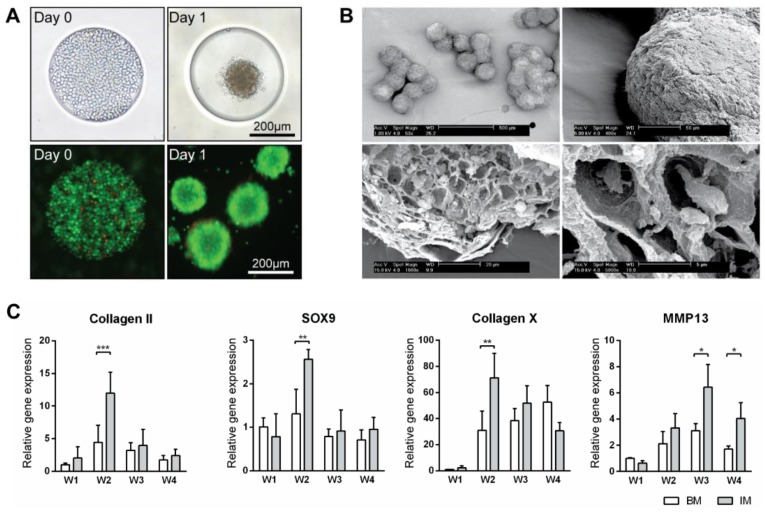
Production of micro-tissue engineered cartilage (MiTEC). Cells were seeded on day 0 and the aggregate formed on day 1; the panel shows brightfield images (top row) and fluorescent images (bottom row) of cells staining with calcein (green) and ethidium homodimer-1 (red) (**A**). Scanning electron microscope images of MiTEC showed uniform size aggregates and cartilage-like lacunae (**B**). Gene expression profile of MiTEC cultured in basic medium (BM—white bar) and basic medium + ITS (IM—gray bar) at week 1–4 (**C**). Error bars represent standard deviation (n = 3). (*) denotes *p* < 0.05, (**) denotes *p* < 0.01, (***) denotes *p* < 0.001.

ATDC5 micro-aggregates were cultured in basic medium (BM) or basic medium supplemented with 1× ITS (IM medium) for 4 weeks on agarose chips. Cell aggregates formed within 24 h after seeding (Figure 1A). Over longer times, the aggregates in IM medium grew bigger and firmer than those in BM medium. After 4 weeks, the aggregates in IM medium developed a very firm appearance whereas those in BM appeared to break down. Scanning electron microscopy of aggregates cultured in IM medium showed a uniform size distribution (Figure 1B). After an even longer culture time, clusters of up to 10 aggregates were observed. The exterior of these aggregates were covered by thick layers of extracellular matrix, while inside them lay many lacunae where the cells resided. Gene expression analysis of the aggregates cultured in BM and IM medium for 4 weeks was performed; expression levels were compared to that of BM medium at week 1 (Figure 1C and Figure S1A). Genes related to early chondrogenesis such as collagen type 2, aggrecan, hypoxia-inducible factors 1 alpha (HIF1α) and Sry-related HMG box (SOX9) as well as late chondrogenesis and hypertrophy such as collagen type 10, matrix metalloproteinases 13 (MMP13), hypoxia-inducible factors 2 alpha (HIF2α) and alkaline phosphatase (ALP) were studied. Expression of all the early markers peaked at week 2 in both BM and IM conditions, although with higher expression in IM medium, then declined in the following weeks. Hypertrophic markers collagen type 10 and ALP expression peaked at week 2, with expression in IM condition double that of the BM condition. MMP13 and HIF2α expression peaked at week 3 and 4 respectively, with expression in the IM always higher than BM condition. This characterization has demonstrated the feasibility of using our spheroid culture system to grow and differentiate ATDC5 in vivo.

### 3.2. Optimization of Chondrogenic Differentiation of MiTEC

**Figure 2:**
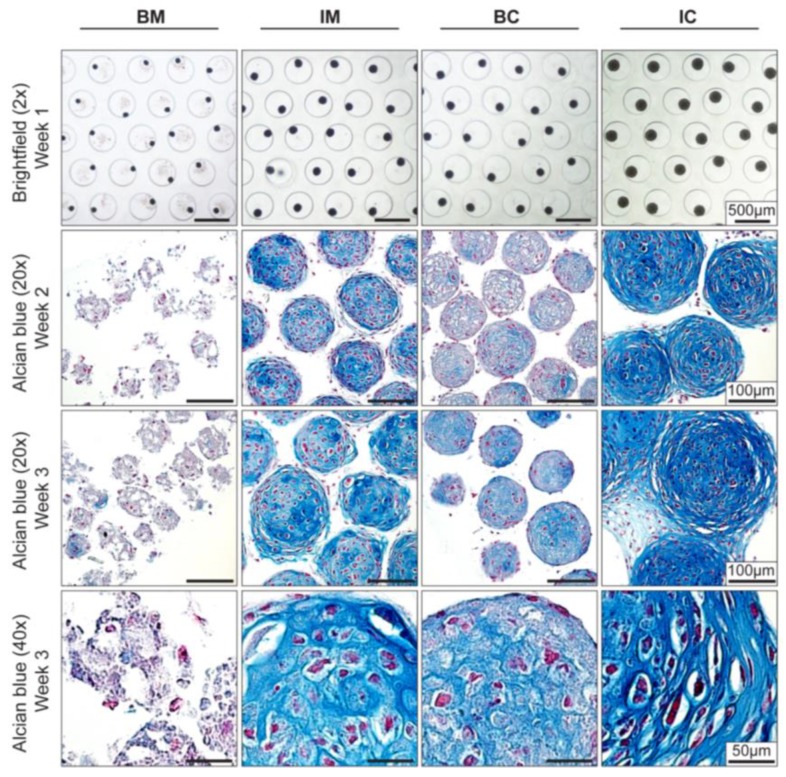
Effect of different media on MiTEC morphology. Aggregates were cultured for 3 weeks in different media: basic medium (**BM**), BM supplemented with 1×ITS (**IM**), BM supplemented with 0.1 μM dexamethasone (Dex) and 10 ng/mL transforming growth factor beta 3 (TGFβ3) (**BC**) and BM supplemented with 1× ITS, 0.1 μM Dex, and 10 ng/mL TGFβ3 (**IC**). Brightfield images at week 1 (**Top Row**) show the difference in aggregate size in which the aggregates in the IC medium was the biggest. Alcian Blue staining at week 2 and 3 (**Row 2**–**4**) shows maturation of the cartilage extracellular matrix; aggregates in the IC medium were stained most intensely and uniformly. Hypertrophic cells can be found at both week 2 and week 3 in the IM, BC and IC medium.

Improved chondrogenic differentiation of aggregates in IM medium showed that it is possible to manipulate the expression of chondrogenic genes. Since the amount of hypertrophic cartilage matrix is known to correlate to in vivo bone formation [6,7], we next maximized the extracellular matrix (ECM) deposition in MiTEC by optimizing medium composition [28]. Four different medium compositions were tested: basic medium (BM); BM supplemented with ITS (IM); BM supplemented with TGFβ3 and Dex (BC); and BM supplemented with ITS, TGFβ3 and Dex (IC). After only 1 week in culture, the difference in size of MiTECs was apparent by light microscopy (Figure 2, top row). In ascending order, BC, IM and IC medium had a positive effect on the size of MiTEC over BM. Histological analysis with Alcian Blue staining for glycosaminoglycan in cartilage (Figure 2) clearly shows that BM was not a good medium to culture MiTEC, with disintegration of the aggregates and very poor Alcian Blue staining. In the IM medium condition, spots with blue stain were visible in week 2 which became bigger and more intense in week 3. Hypertrophic chondrocytes, recognized by their large lacunae, appeared to reside within the intensely blue stained extracellular matrix. Interestingly, the use of both TGFβ3 and Dex (BC condition) resulted only in a faint Alcian Blue stain at both week 2 and 3. The aggregates in the BC condition were also smaller in size compared to the IM condition (just ITS added to the basic medium). Finally, when cultured in the presence of TGFβ3, Dex and ITS (IC condition), a uniform and intense blue stain was observed in all aggregates.

**Figure 3:**
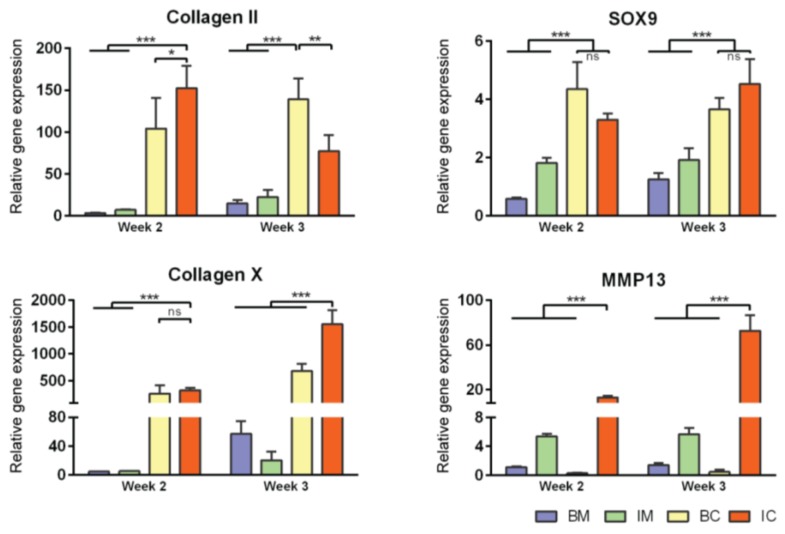
Effect of different media on MiTEC gene expression. Gene expression analysis of aggregates cultured in basic medium (BM—blue bar), BM + ITS (IM—green bar), BM + TGFβ + Dex (BC—yellow bar), and BM + ITS + TGFβ + Dex (IC—red bar) at week 2–3. Error bars represent standard deviation (n = 3). (*) denotes *p* < 0.05, (**) denotes *p* < 0.01, (***) denotes *p* < 0.001, and ns denotes non-significant.

Gene expression analysis for early and late chondrogenic markers at week 2 and 3 was performed; expression levels were compared to that of BM at week 2 (Figure 3 and Figure S1B). For early markers (collagen type 2, aggrecan, SOX9, and HIF1α), gene expression in BC and IC conditions (both contained TGFβ3 and Dex) was always higher than those of BM and IM conditions (except for HIF1α where the expression in the BC condition was similar to those of BM and IM). For late markers, collagen type 10 expression in the BC and IC conditions was particularly high (300 times at week 2 and 500–1500 times at week 3) compared to BM and IM. MMP13 expression was only high in the IM and IC conditions (both contained ITS), 5-12 times at week 2 and 5–70 times at week 3 compared to the BM condition respectively. Interestingly, HIF2α, ALP and VEGF expression was highest in the IM condition at both week 2 and 3. Overall, in term of aggregate size, extracellular matrix stain for Alcian Blue and certain gene markers, IC is the most potent medium to induce extracellular matrix deposition and hypertrophic differentiation of MiTEC. Thus we chose the IC medium to continue with for the next experiments.

### 3.3. Induction of MiTEC Mineralization with Beta-Glycerophosphate

**Figure 4:**
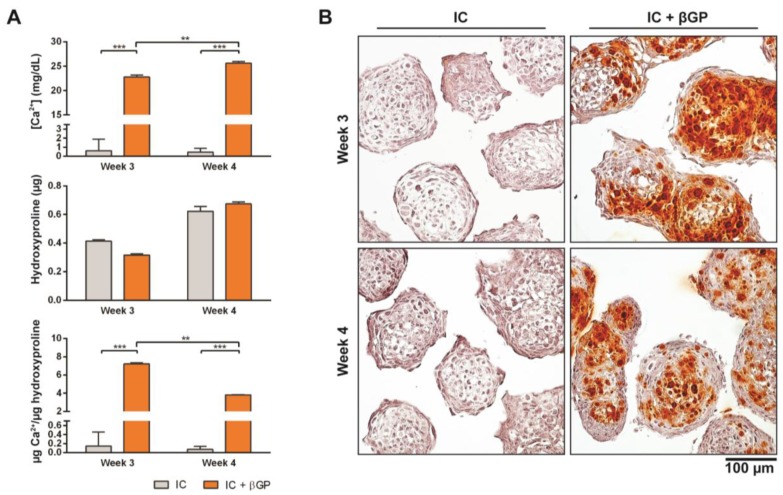
Induction of MiTEC mineralization with beta-glycerophosphate. Calcium content, hydroxyproline content and calcium content per µg of hydroxyproline of aggregates cultured in IC medium for 2 or 3 weeks, plus 1 week treatment with beta-glycerophosphate (bGP) (**A**). Error bars represent the standard deviation (n = 3). (**) denotes *p* < 0.01, and (***) denotes *p* < 0.001. Alizarin Red staining of aggregates cultured in IC medium for 2 or 3 weeks, plus 1 week treatment with bGP; red nodules can be seen homogeneously inside the aggregates (**B**).

Just prior to endochondral ossification, end stage hypertrophic chondrocytes direct the formation of mineralized matrix, which becomes a scaffold for osteoprogenitor cells to interact with and secrete osteoid [31]. Pre-mineralizing the hypertrophic cartilage construct in vivo may improve and accelerate bone formation upon in vivo implantation. In this experiment, we induced in vivo mineralization of MiTECs with beta-glycerophosphate (bGP). MiTECs were cultured for 2 or 3 weeks in IC medium, followed by 1 week in mineralization medium with 10 mM bGP. MiTECs were collected at week 3 (2 weeks in IC medium plus 1 week in mineralization medium) and week 4 (3 weeks in IC medium plus 1 week in mineralization medium) for analysis. Calcium analysis showed a significant increase in the calcium content of MiTECs cultured in the mineralization medium (Figure 4A). Hydroxyproline content (estimated organic content of the extracellular matrix) increased from week 3–4 but was not affected by the bGP treatment. Histological analysis with Alizarin Red staining showed intense red nodules in the bGP condition (Figure 4B). Gene expression analysis was performed for the hypertrophic markers HIF2α, ALP, VEGF, collagen 10 and MMP13 (Figure S2A). Interestingly, the addition of bGP increased HIF2α, VEGF, collagen X and MMP13 expression at week 4 but not at week 3 of the culture. Expression of ALP after 1 week in mineralization medium either decreased (week 3) or was unchanged (week 4). Overall, the in vivo mineralization of MiTEC could be efficiently achieved with the bGP treatment, which may add an edge to the in vivo performance of the material.

### 3.4. Boosting Vascular Endothelial Growth Factor (VEGF) Secretion from MiTEC Using the Hypoxia Mimetic Phenanthroline

**Figure 5:**
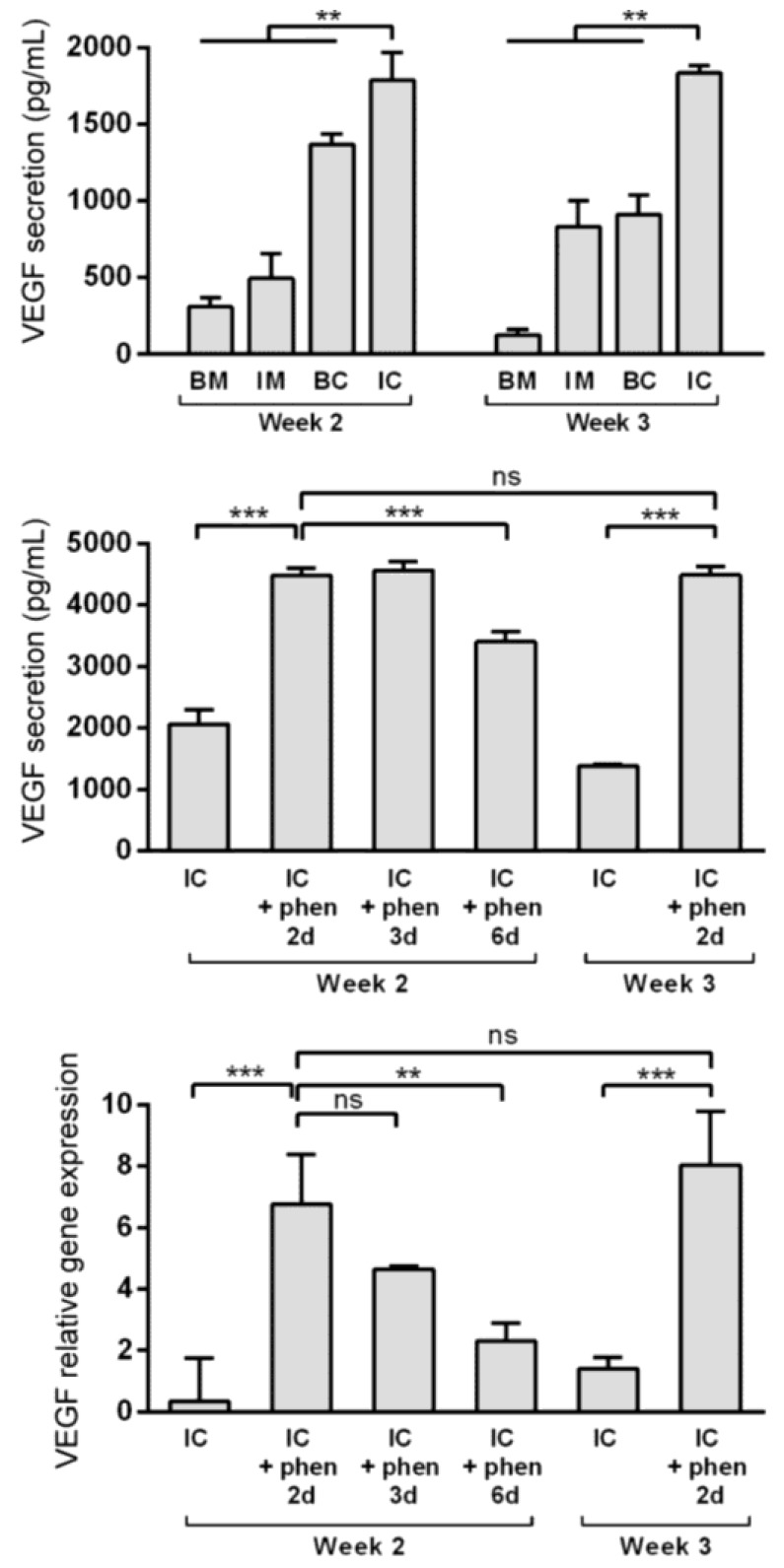
Boosting vascular endothelial growth factor (VEGF) expression and secretion from MiTEC using the hypoxia mimetic phenanthroline. VEGF secretion of aggregates cultured in BM (blue bar), IM (green bar), BC (yellow bar) and IC (red bar) medium at week 2–3 (top graph). VEGF secretion (middle graph) and expression (bottom graph) of aggregates cultured in IC medium for 2 or 3 weeks followed by phenanthroline treatment for 2, 3 or 6 days. Error bars represent standard deviation (n = 3). (**) denotes *p* < 0.01, (***) denotes *p* < 0.001, and ns denotes non-significant.

Vascular invasion of the hypertrophic cartilage is a crucial event in endochondral bone formation and VEGF plays a critical role in it [32,33]. Enriching the extracellular matrix with VEGF may improve MiTEC performance. Previously, using high throughput screening, we identified phenanthroline as a hypoxia mimicking molecule capable of inducing VEGF expression and secretion in hMSCs [30]. Preliminary experiments were done to verify that phenanthroline can also stimulate VEGF expression and secretion of ATDC5 cells cultured in a monolayer (data not shown). In the aggregate culture, VEGF expression was highest in the IM medium (Figure S1B), but VEGF secretion as detected in the medium was highest in the IC medium at about 2000 pg/mL (Figure 5, top graph). To test the effect of phenanthroline on MiTEC, we cultured the aggregates for 2 or 3 weeks in IC medium, followed by a 2, 3 or 6 day exposure to 50 µM phenanthroline. Phenanthroline induced a 7-fold induction of VEGF expression after a 2 day exposure at week 2 of the culture (Fig 5, bottom graph). However, this expression reduced if the exposure was prolonged to 3 days and 6 days. Exposing MiTEC with phenanthroline for 2 days at week 3 of the culture also resulted in an 8-fold increase in the mRNA level. The VEGF secretion baseline at 2000 pg/mL in the IC medium was more than doubled when phenanthroline was added for 2 days at week 2 of the culture (Figure 5, middle graph). However, when phenanthroline treatment was extended to 6 days, the secretion reduced to about 3000 pg/mL. A 2-day stimulation in week 3 resulted in the same secretion level as in week 2. Thus, using phenanthroline, we could efficiently boost VEGF expression and secretion from MiTEC.

### 3.5. Devitalization and Decellularization of MiTEC

**Figure 6:**
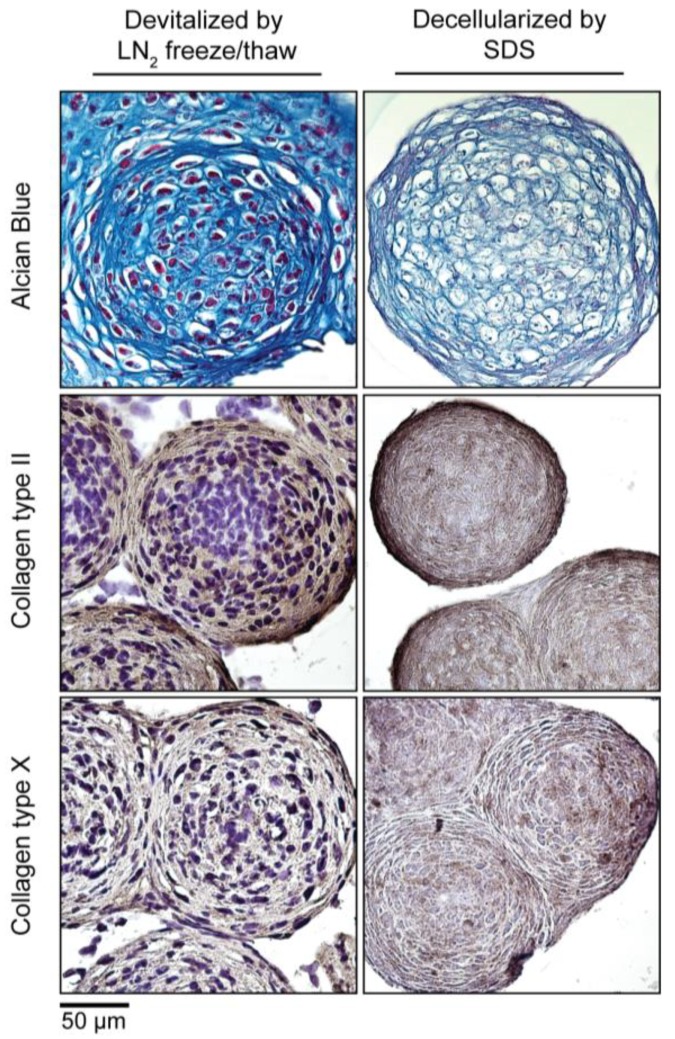
Devitalization and decellularization of MiTEC. Aggregates were cultured for 2 weeks in IC medium, and then were either devitalized by repeated liquid nitrogen (LN2) freeze/thaw or decellularized by SDS. Picture panel shows Alcian Blue staining (**Top Row**); collagen type 2 staining (**Middle Row**); and collagen type 10 staining (**Bottom Row**).

To make MiTEC an off-the-shelf product, devitalization/decellularization is necessary in order to reduce the immunogenicity of the material. We devitalized MiTEC by liquid nitrogen (LN2) freeze-thawing and decellularized MiTEC by SDS processing following established protocols. Devitalized/decellularized MiTECs were tested for cell survival by a metabolism assay where no metabolic activity was found (data not shown). The amount of MiTECs harvested from one 12-well plate, decellularized with SDS and air dried is shown in Fig S2B. The average diameter of an aggregate after a 3-week culture in IC medium was 300 µm (Figure 6). Assuming each aggregate is a sphere; its volume is 43π×1503=14,137,167 µm3 or 0.014 mm^3^. Each well of the 12-well plate containing 1585 aggregates yielded 22.4 mm^3^, and one 12-well plate yielded 268.9 mm^3^ of tissue. Four culture plates would be needed to make 1CC of tissue. Histological analysis with Alcian Blue and immunostaining for collagen type 2 and type X is shown in Figure 6. No cell nuclei were visible in the histological images of the decellularized MiTEC. Alcian Blue staining was reduced significantly in the decellularized samples, however collagen type 2 and X staining were similar to that of the devitalized samples. Collagen type 2 staining was more eminent at the periphery of the aggregates, while collagen type 10 was distributed more uniformly.

### 3.6. DCM Influences Chondrogenic Differentiation of hMSCs

**Figure 7:**
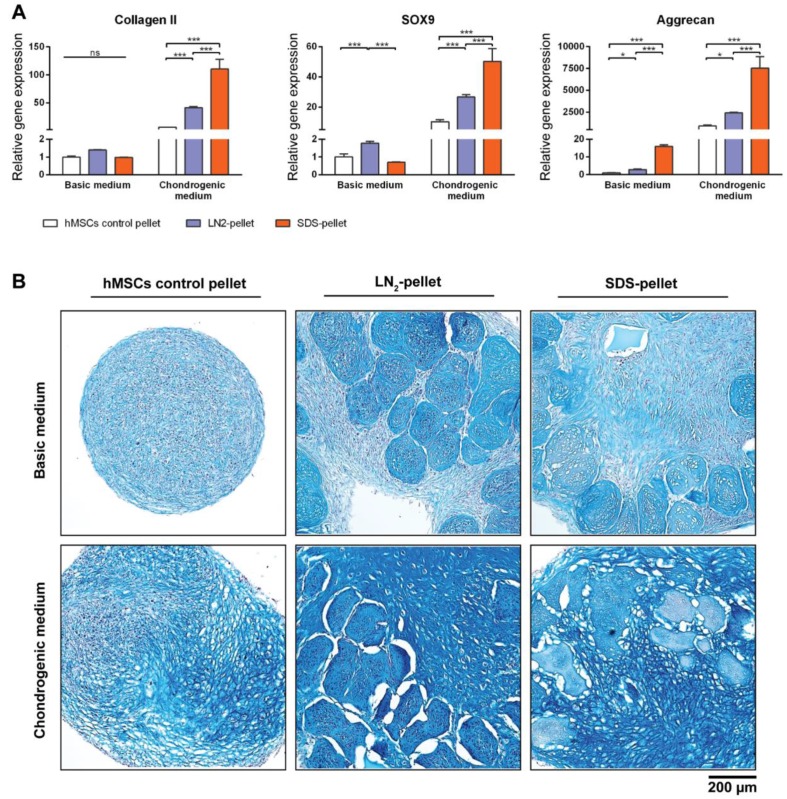
MiTEC affects chondrogenic differentiation of human mesenchymal stem cells (hMSCs). Gene expression analysis of hMSCs co-cultured with devitalized or decellularized MiTEC in basic medium or chondrogenic medium (**A**). Error bars represent standard deviation (n = 3). (*) denotes *p* < 0.05, (**) denotes *p* < 0.01, (***) denotes *p* < 0.001, and ns denotes non-significant. Alcian Blue staining of hMSCs co-cultured with devitalized or decellularized MiTEC in basic medium (top row) or chondrogenic medium (bottom row) (**B**).

To test the biological properties of our DCM, devitalized/decellularized MiTECs were mixed with hMSCs and pellet-cultured for 4 weeks in either basic medium or chondrogenic differentiation medium. Pellets of hMSCs mixed with LN2-devitalized MiTEC are referred to as LN2-pellets, and pellets of hMSCs mixed with SDS-decellularized MiTEC are referred to as SDS-pellets. As a control, hMSCs were cultured in a pellet of 250,000 cells which is the same number of cells used in the mix-culture with MiTEC. After 4 weeks, the LN2-pellets and SDS-pellets cultured in chondrogenic medium developed a glassy appearance and felt very firm (Figure S2C). Gene expression analysis was performed for early chondrogenesis makers collagen type 2, aggrecan and SOX9 (Figure 7A). Relative to hMSC pellets, expression of these markers increased significantly in the SDS-pellet in chondrogenic medium. Even in basic medium, aggrecan expression increased 20-fold in the SDS-pellet compared to the hMSCs pellet. Expression of the 3 markers in the LN2-pellet was also higher than hMSC alone control, but lower than the SDS-pellets. Alcian Blue staining showed that the devitalized/decellularized MiTECs were still visible and embedded inside the cell pellets (Figure 7B). No cell was found inside the SDS treated MiTECs. In basic medium, beside the intense blue stain of the devitalized MiTEC aggregates, the rest of the pellet was weakly stained. In chondrogenic differentiation medium, the hMSCs alone control developed some blue stains only in some parts of the pellets. However, when hMSCs were co-cultured with either LN2 or SDS treated MiTEC, Alcian Blue was intensely stained throughout the pellets. The MiTEC appeared to be shrunken inside the pellets under the chondrogenic medium condition, with a big gap between MiTEC and the human cells. With this experiment, we showed that devitalized/decellularized MiTEC has chondro-inductive potential in vivo.

## 4. Discussion

Although autologous bone is considered as the most superior grafting material for bone regeneration [34], other bone graft substitutes are increasingly preferred not only for the convenience of the surgeons but also for the good of the patients [35]. In nature, bone healing occurs through both endochondral and intramembranous ossification [36]. While intramembranous ossification is the fastest route of building bone matrix, it can only handle small and mechanically stable defects [36,37]. Endochondral ossification, while it is slow and requires a lot more energy, is the predominant route of bone healing in fractures. During the process, mesenchymal progenitor cells differentiate into chondrocytes which deposit cartilaginous matrix called the soft callus. In animal models, the soft callus formation peaks at 7-9 days post trauma with a peak in collagen type 2 and proteoglycan such as aggrecan [38]. The chondrocytes of the soft callus undergo maturation towards hypertrophy, becoming enlarged in size, and secreting alkaline phosphatase which mineralizes the extracellular matrix. The fate of the hypertrophic chondrocytes remains controversial. Classically, the hypertrophic chondrocytes undergo apoptosis and the osteoprogenitor cells invade the callus and replace the cartilage with bone. Alternatively, studies have shown that a proportion of hypertrophic chondrocytes do not undergo apoptosis and instead transdifferentiate into osteoblasts [12,39]. In the classical model, the role of hypertrophic chondrocytes is less important after its accomplishment of synthesizing the necessary growth factors. This suggests that a hypertrophic cartilage matrix void of cells but high in endogenous growth factors can continue its route to be transformed into bone in vivo. To date, no clinical case using hypertrophic cartilage for bone regeneration has been reported. Despite abundant proof-of-principle studies, no available method can yet produce sufficient hypertrophic cartilage for clinical use. Here we demonstrated that by using the cell line ATDC5 cultured in micro-aggregates, it was feasible to produce hypertrophic cartilage in unlimited amounts. According to the FDA’s guideline for clinical issues concerning the use of xenotransplantation products in humans [40], “cell lines from animals may be established and used in the production of xenotransplantation products”. Compared to primary cells, cell lines are easier to maintain and more consistent in quality. Since cell lines are carcinogenic, devitalization of the hypertrophic cartilage is necessary to reduce immunogenic concerns. Whether or not the devitalized MiTEC is still capable of inducing endochondral ossification in vivo needs further experiments. Bourgine et al. demonstrated that apoptosis-induced devitalized hypertrophic cartilage constructs could form bone in vivo while freeze-thawed devitalized constructs could not [18]. They attributed this to the significant loss of glycosaminoglycans, mineral content, and ECM-bound cytokines during the freeze-thaw cycles. We also observed a loss of glycosaminoglycans, as indicated by Alcian Blue staining, in the SDS decellularized MiTEC, but not in the freeze-thaw devitalized MiTEC. Apparently, the bioactivity of the hypertrophic cartilage ECM may be lost when a sub-optimal devitalization/decellularization process is used. Hence, the balance of removing immunogenicity and retaining osteoinductivity needs to be fine-tuned, for which many devitalization/decellularization methods have been reported [41,42,43]. Our model can potentially be adapted into a high throughput screening platforms in vivo and in vivo, allowing the devitalization/decellularization protocol to be empirically optimized.

Vascularization is a critical factor for the successful integration of large grafts. Hypertrophic cartilage has an intrinsic property of produce angiogenic growth factors, which play a crucial role in endochondral ossification and many studies have shown the positive effect of adding VEGF in bone regeneration [33,44]. It has been shown that the lack of vascularization in the DCM constructs could be attributed to the loss of VEGF during the devitalization process [18]. Vascularization in the case of endochondral bone formation is essential for the recruitment of the host cells in order to remodel the cartilage template and subsequent ossification. We showed that stimulating MiTEC with the hypoxia mimicking molecule phenanthroline boosted VEGF secretion to double its basal level, although the VEGF content which remained in the decellularized MiTEC has not been analyzed. Since vascularization is paramount to the success of this model, it may be necessary to further optimize it for instance by combining it with current vascularization strategies, i.e., VEGF impregnation [45]. Similarly, pre-mineralization of MiTEC in vivo may be beneficial for bone formation in vivo. Calcium phosphate ceramics have a positive effect on osteogenesis [46] and even soluble calcium ions have a positive effect on the osteogenic differentiation of hMSCs [47]. The degree of mineralization is another aspect which can be optimized for bone formation in vivo.

The inductive potential of decellularized ECM on stem cells [48,49] has been reported. It was hypothesized that the acellular ECM of a tissue possessed the instructive cues that drive the differentiation of stem cells into this particular tissue type [50,51]. The instructive cues are the soluble factors and the macromolecules that regulate cell fate. To date, no study has demonstrated the inductive potential of hypertrophic cartilage matrix in vivo. In this paper, we show that devitalized/decellularized MiTEC, especially in combination with chondrogenic medium, enhanced the chondrogenic differentiation of hMSCs in vivo. From histological images it appeared that the MiTEC ECM remained intact and hMSCs were not able to enter the cartilage matrix. Thus, it’s arguable that the inductive potential of devitalized/decellularized MiTEC is caused by the soluble factors released from it, although the surface chemistry and topography of the tissue might also play a role. Interestingly, SDS decellularized MiTEC appeared to be more potent than the freeze-thaw devitalized aggregates. This may be similar to the biological activity of bone versus demineralized bone, where the removal of bone mineral makes the osteogenic molecules available for interaction with the cells [52].

Unlike natural products harvested from cadavers where donor variation is inevitable, hypertrophic cartilage culture in vivo is a totally controllable process. There are many concerns over the variation in quality among allogenic bone grafts produced by different companies or even from different batches of product from the same company [53,54]. Moreover, some products such as DBM and platelet gels containing “autologous growth factors” are not subjected to high level of regulatory scrutiny [55]. In contrast, MiTEC can be cultured and tested for both safety and efficacy following stringent good manufacturing practice (GMP) protocols. Here, we demonstrated how MiTEC can be manipulated in a number of ways to modify its extracellular matrix composition. The well plate format of this system allows for high throughput screening to be used in drug discovery and, using a different cell source or cell sources, one can even switch this model to the production of a totally different type of tissue, for instance to produce liver tissue or to study tumor cell biology. In conclusion, we have shown a method to produce clinically relevant-sized hypertrophic cartilage for bone regeneration by endochondral ossification. We decellularized the hypertrophic tissue and showed that the remaining ECM had inductive potential on chondrogenic differentiation of hMSCs in vivo. The logical next phase of this work is the pre-clinical evaluation of MiTEC in orthotopic models.

## 5. Conclusions

The results of this study demonstrate the feasibility of producing in large amounts and to readily vary the chemical composition of hypertrophic cartilage tissue in vivo using the ATDC5 cell line cultured in micro-aggregates (MiTEC). Moreover, under appropriate culturing and devitalizing conditions, the devitalized MiTEC can enhance chondrogenic differentiation of hMSCs, suggesting that devitalized MiTEC represents a promising advance for clinical applications in bone regeneration. 

## Figures and Tables

**Table 1:** Primer sequence.

Name	Primer Sequence
Mouse beta-2 microglobulin	5′-CATGGCTCGCTCGGTGACC-3′
	5′- AATGTGAGGCGGGTGGAACTG-3′
Mouse collagen 2 alpha	5′-CAAGGCCCCCGAGGTGACAAA-3′
	5′-GGGGCCAGGGATTCCATTAGAGC-3′
Mouse collagen 10 alpha	5′-CATAAAGGGCCCACTTGCTA-3′
	5′-TGGCTGATATTCCTGGTGGT-3′
Mouse aggrecan	5′-AGAACCTTCGCTCCAATGACTC-3′
	5′-AGGGTGTAGCGTGTGGAAATAG-3′
Mouse Sry-related HMG box 9 (SOX9)	5′-CCACGGAACAGACTCACATCTCTC-3′
	5′-CTGCTCAGTTCACCGATGTCCACG-3′
Mouse hypoxia-inducible factors 1 alpha (HIF1α)	5′-TGCTCATCAGTTGCCACTTC-3′
	5′-TGGGCCATTTCTGTGTGTAA-3′
Mouse hypoxia-inducible factors 2 alpha (HIF2α)	5′-TGAGTTGGCTCATGAGTTGC-3′
	5′-CTCACGGATCTCCTCATGGT-3′
Mouse alkaline phosphatase (ALP)	5′-AACCCAGACACAAGCATTCC-3′
	5′-GAGACATTTTCCCGTTCACC-3′
Mouse matrix metalloproteinases 13 (MMP13)	5′-AGGCCTTCAGAAAAGCCTTC-3′
	5′-TCCTTGGAGTGATCCAGACC-3′
Human B2M	5′-GACTTGTCTTTCAGCAAGGA-3′
	5′-ACAAAGTCACATGGTTCACA-3′
Human collagen 2 alpha	5′-CGTCCAGATGACCTTCCTACG-3′
	5′-TGAGCAGGGCCTTCTTGAG-3′
Human aggrecan	5′-AGAATCCACCACCACCAG-3′
	5′-ATGCTGGTGCTGATGACA-3′
Human SOX9	5′-TGGGCAAGCTCTGGAGACTTC-3′
	5′-ATCCGGGTGGTCCTTCTTGTG-3′

## Supplementary Materials

The supplementary materials are available online at http://www.mdpi.com/2306-5354/4/2/35/s1.

## References

[1] Ferguson C., Alpern E., Miclau T., Helms J.A. (1999). Does adult fracture repair recapitulate embryonic skeletal formation?. Mech. Dev..

[2] Einhorn T.A., Gerstenfeld L.C. (2015). Fracture healing: Mechanisms and interventions. Nat. Rev. Rheumatol..

[3] Gerstenfeld L.C., Cullinane D.M., Barnes G.L., Graves D.T., Einhorn T.A. (2003). Fracture healing as a post-natal developmental process: Molecular, spatial, and temporal aspects of its regulation. J. Cell. Biochem..

[4] Durao S.F., Gomes P.S., Silva-Marques J.M., Fonseca H.R., Carvalho J.F., Duarte J.A., Fernandes M.H. (2012). Bone regeneration in osteoporotic conditions: Healing of subcritical-size calvarial defects in the ovariectomized rat. Int. J. Oral Maxillofac. Implants.

[5] Dinopoulos H., Dimitriou R., Giannoudis P.V. (2012). Bone graft substitutes: What are the options?. Surg. J. R. Coll. Surg. Edinb. Irel..

[6] Jukes J.M., Both S.K., Leusink A., Sterk L.M., van Blitterswijk C.A., de Boer J. (2008). Endochondral bone tissue engineering using embryonic stem cells. Proc. Natl. Acad. Sci. USA.

[7] Scotti C., Tonnarelli B., Papadimitropoulos A., Scherberich A., Schaeren S., Schauerte A., Lopez-Rios J., Zeller R., Barbero A., Martin I. (2010). Recapitulation of endochondral bone formation using human adult mesenchymal stem cells as a paradigm for developmental engineering. Proc. Natl. Acad. Sci. USA.

[8] Weiss H.E., Roberts S.J., Schrooten J., Luyten F.P. (2012). A semi-autonomous model of endochondral ossification for developmental tissue engineering. Tissue Eng. Part A.

[9] Farrell E., Both S.K., Odorfer K.I., Koevoet W., Kops N., O’Brien F.J., Baatenburg de Jong R.J., Verhaar J.A., Cuijpers V., Jansen J. (2011). In-vivo generation of bone via endochondral ossification by in-vitro chondrogenic priming of adult human and rat mesenchymal stem cells. BMC Musculoskelet. Disord..

[10] Gawlitta D., Farrell E., Malda J., Creemers L.B., Alblas J., Dhert W.J. (2010). Modulating endochondral ossification of multipotent stromal cells for bone regeneration. Tissue Eng. Part B Rev..

[11] Scotti C., Piccinini E., Takizawa H., Todorov A., Bourgine P., Papadimitropoulos A., Barbero A., Manz M.G., Martin I. (2013). Engineering of a functional bone organ through endochondral ossification. Proc. Natl. Acad. Sci. USA.

[12] Bahney C.S., Hu D.P., Taylor A.J., Ferro F., Britz H.M., Hallgrimsson B., Johnstone B., Miclau T., Marcucio R.S. (2014). Stem cell-derived endochondral cartilage stimulates bone healing by tissue transformation. J. Bone Miner. Res..

[13] Urist M.R. (1965). Bone: Formation by autoinduction. Science.

[14] Bridges J.B., Pritchard J.J. (1958). Bone and cartilage induction in the rabbit. J. Anat..

[15] Urist M.R., Mc L.F. (1952). Osteogenetic potency and new-bone formation by induction in transplants to the anterior chamber of the eye. J. Bone Jt. Surg. Am..

[16] Urist M.R., Wallace T.H., Adams T. (1965). The function of fibrocartilaginous fracture callus. Observations on transplants labelled with tritiated thymidine. J. Bone Jt. Surg. Br..

[17] Urist M.R., Adams T. (1968). Cartilage or bone induction by articular cartilage. Observations with radioisotope labelling techniques. J. Bone Jt. Surg. Br..

[18] Bourgine P.E., Scotti C., Pigeot S., Tchang L.A., Todorov A., Martin I. (2014). Osteoinductivity of engineered cartilaginous templates devitalized by inducible apoptosis. Proc. Natl. Acad. Sci. USA.

[19] Cunniffe G.M., Vinardell T., Murphy J.M., Thompson E.M., Matsiko A., O’Brien F.J., Kelly D.J. (2015). Porous decellularized tissue engineered hypertrophic cartilage as a scaffold for large bone defect healing. Acta Biomater..

[20] Jukes J.M., Moroni L., van Blitterswijk C.A., de Boer J. (2008). Critical steps toward a tissue-engineered cartilage implant using embryonic stem cells. Tissue Eng. Part A.

[21] Siddappa R., Licht R., van Blitterswijk C., de Boer J. (2007). Donor variation and loss of multipotency during in vivo expansion of human mesenchymal stem cells for bone tissue engineering. J. Orthop. Res..

[22] Yao Y., Wang Y. (2013). Atdc5: An excellent in vivo model cell line for skeletal development. J. Cell. Biochem..

[23] Altaf F.M., Hering T.M., Kazmi N.H., Yoo J.U., Johnstone B. (2006). Ascorbate-enhanced chondrogenesis of atdc5 cells. Eur. Cells Mater..

[24] Caron M.M., Emans P.J., Cremers A., Surtel D.A., Coolsen M.M., van Rhijn L.W., Welting T.J. (2013). Hypertrophic differentiation during chondrogenic differentiation of progenitor cells is stimulated by bmp-2 but suppressed by bmp-7. Osteoarthr. Cartil..

[25] Rivron N.C., Vrij E.J., Rouwkema J., Le Gac S., van den Berg A., Truckenmuller R.K., van Blitterswijk C.A. (2012). Tissue deformation spatially modulates vegf signaling and angiogenesis. Proc. Natl. Acad. Sci. USA.

[26] Fennema E., Rivron N., Rouwkema J., van Blitterswijk C., de Boer J. (2013). Spheroid culture as a tool for creating 3d complex tissues. Trends Biotechnol..

[27] Alves H., Mentink A., Le B., van Blitterswijk C.A., de Boer J. (2013). Effect of antioxidant supplementation on the total yield, oxidative stress levels, and multipotency of bone marrow-derived human mesenchymal stromal cells. Tissue Eng. Part A.

[28] Mackay A.M., Beck S.C., Murphy J.M., Barry F.P., Chichester C.O., Pittenger M.F. (1998). Chondrogenic differentiation of cultured human mesenchymal stem cells from marrow. Tissue Eng..

[29] Kheir E., Stapleton T., Shaw D., Jin Z., Fisher J., Ingham E. (2011). Development and characterization of an acellular porcine cartilage bone matrix for use in tissue engineering. J. Biomed. Mater. Res. Part A.

[30] Doorn J., Fernandes H.A., Le B.Q., van de Peppel J., van Leeuwen J.P., De Vries M.R., Aref Z., Quax P.H., Myklebost O., Saris D.B. (2013). A small molecule approach to engineering vascularized tissue. Biomaterials.

[31] Kronenberg H.M. (2003). Developmental regulation of the growth plate. Nature.

[32] Dai J., Rabie A.B. (2007). Vegf: An essential mediator of both angiogenesis and endochondral ossification. J. Dent. Res..

[33] Yang Y.Q., Tan Y.Y., Wong R., Wenden A., Zhang L.K., Rabie A.B. (2012). The role of vascular endothelial growth factor in ossification. Int. J. Oral Sci..

[34] Pape H.C., Evans A., Kobbe P. (2010). Autologous bone graft: Properties and techniques. J. Orthop. Trauma.

[35] Parikh S.N. (2002). Bone graft substitutes: Past, present, future. J. Postgrad. Med..

[36] Marsell R., Einhorn T.A. (2011). The biology of fracture healing. Injury.

[37] Shapiro F. (2008). Bone development and its relation to fracture repair. The role of mesenchymal osteoblasts and surface osteoblasts. Eur. Cells Mater..

[38] Einhorn T.A. (1998). The cell and molecular biology of fracture healing. Clin. Orthop. Relat. Res..

[39] Hu D.P., Ferro F., Yang F., Taylor A.J., Chang W., Miclau T., Marcucio R.S., Bahney C.S. (2017). Cartilage to bone transformation during fracture healing is coordinated by the invading vasculature and induction of the core pluripotency genes. Development.

[40] Food and Drug Administration (2011). Guidance for Industry: Source animal, Product, Preclinical, and Clinical Issues Concerning the Use of Xenotransplantation Products in Humans.

[41] Lu H., Hoshiba T., Kawazoe N., Chen G. (2012). Comparison of decellularization techniques for preparation of extracellular matrix scaffolds derived from three-dimensional cell culture. J. Biomed. Mater. Res. Part A.

[42] Crapo P.M., Gilbert T.W., Badylak S.F. (2011). An overview of tissue and whole organ decellularization processes. Biomaterials.

[43] Dong J., Mo X., Li Y., Chen D. (2012). [recent research progress of decellularization of native tissues]. Sheng Wu Yi Xue Gong Cheng Xue Za Zhi.

[44] Aryal R., Chen X.P., Fang C., Hu Y.C. (2014). Bone morphogenetic protein-2 and vascular endothelial growth factor in bone tissue regeneration: New insight and perspectives. Orthop. Surg..

[45] Krishnan L., Willett N.J., Guldberg R.E. (2014). Vascularization strategies for bone regeneration. Ann. Biomed. Eng..

[46] Zhang J., Luo X., Barbieri D., Barradas A.M., de Bruijn J.D., van Blitterswijk C.A., Yuan H. (2014). The size of surface microstructures as an osteogenic factor in calcium phosphate ceramics. Acta Biomater..

[47] Barradas A.M., Fernandes H.A., Groen N., Chai Y.C., Schrooten J., van de Peppel J., van Leeuwen J.P., van Blitterswijk C.A., de Boer J. (2012). A calcium-induced signaling cascade leading to osteogenic differentiation of human bone marrow-derived mesenchymal stromal cells. Biomaterials.

[48] Hoganson D.M., Meppelink A.M., Hinkel C.J., Goldman S.M., Liu X.H., Nunley R.M., Gaut J.P., Vacanti J.P. (2014). Differentiation of human bone marrow mesenchymal stem cells on decellularized extracellular matrix materials. J. Biomed. Mater. Res. Part A.

[49] Guo Y., Zeng Q., Yan Y., Shen L., Liu L., Li R., Zhang X., Wu J., Guan J., Huang S. (2013). Proliferative effect and osteoinductive potential of extracellular matrix coated on cell culture plates. SpringerPlus.

[50] Brown B.N., Badylak S.F. (2014). Extracellular matrix as an inductive scaffold for functional tissue reconstruction. Transl. Res..

[51] Reilly G.C., Engler A.J. (2010). Intrinsic extracellular matrix properties regulate stem cell differentiation. J. Biomech..

[52] Zhang M., Powers R.M., Wolfinbarger L. (1997). A quantitative assessment of osteoinductivity of human demineralized bone matrix. J. Periodontol..

[53] Aghdasi B., Montgomery S.R., Daubs M.D., Wang J.C. (2013). A review of demineralized bone matrices for spinal fusion: The evidence for efficacy. Surg. J. R. Coll. Surg. Edinb. Irel..

[54] Bae H.W., Zhao L., Kanim L.E., Wong P., Delamarter R.B., Dawson E.G. (2006). Intervariability and intravariability of bone morphogenetic proteins in commercially available demineralized bone matrix products. Spine.

[55] Greenwald A.S., Boden S.D., Goldberg V.M., Khan Y., Laurencin C.T., Rosier R.N. (2001). Bone-graft substitutes: Facts, fictions, and applications. J. Bone Jt. Surg. Am..

