# Supplementary material for: An Approach to In Vitro Manufacturing of Hypertrophic Cartilage Matrix for Bone Repair
Source: Bioengineering (Basel). 2017 Apr 20;4(2):35. doi: 10.3390/bioengineering4020035 (PMC5590482; doi:10.3390/bioengineering4020035)
Supplement: Supplementary file 1 [file bioengineering-04-00035-s001.pdf]

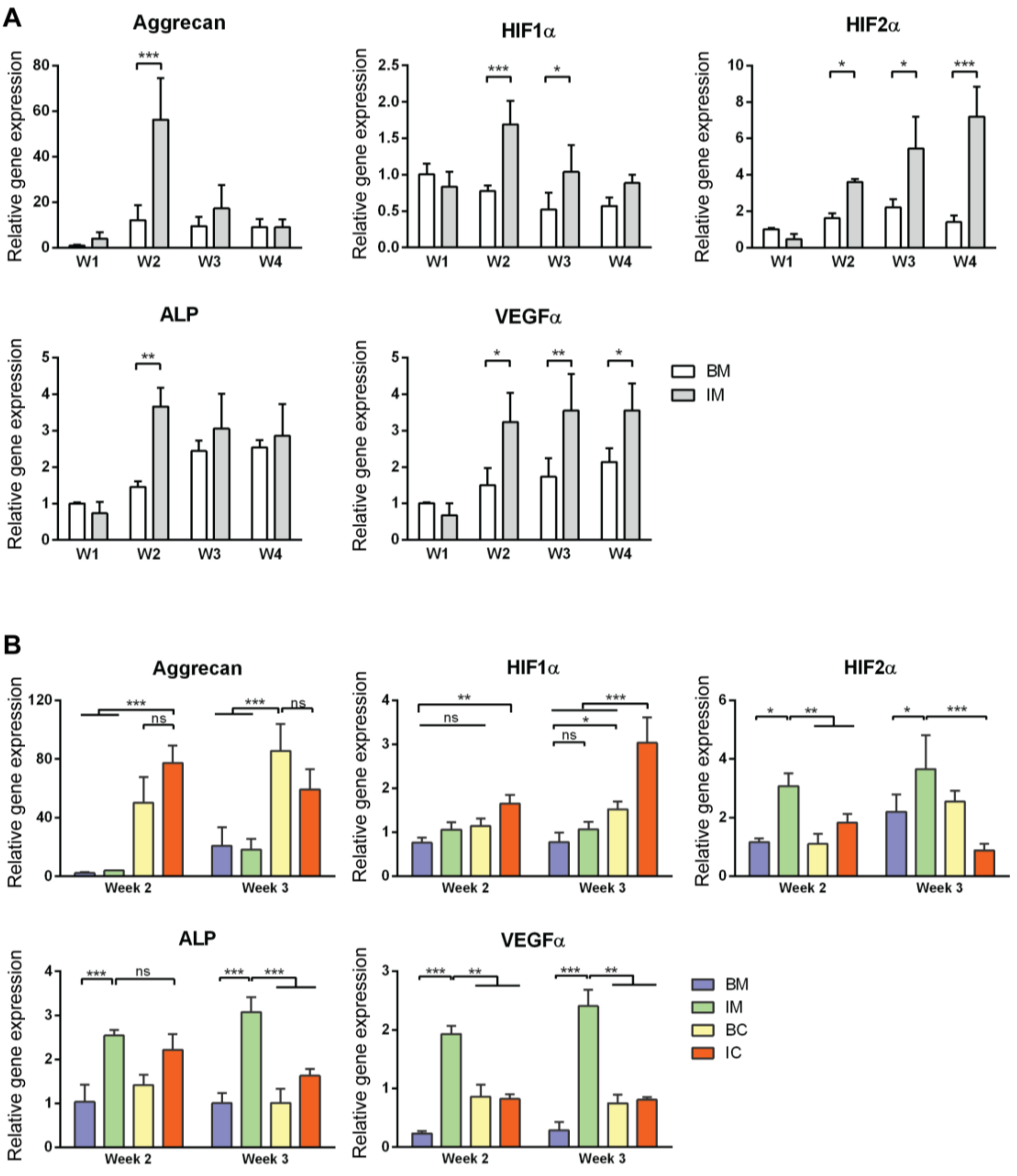

Figure S1. Gene expression of MiTEC cultured in BM (white bar) and IM (gray bar) at week 1-4 **(A)**. Gene expression of MiTEC cultured in BM (blue bar), IM (green bar), BC (yellow bar) and IC (red bar) medium at week 2-3 **(B)**. Error bars represent standard deviation (n=3). (*) denotes $p<0.05$, (**) denotes $p<0.01$, (***) denotes $p<0.001$, and ns denotes non-significant.

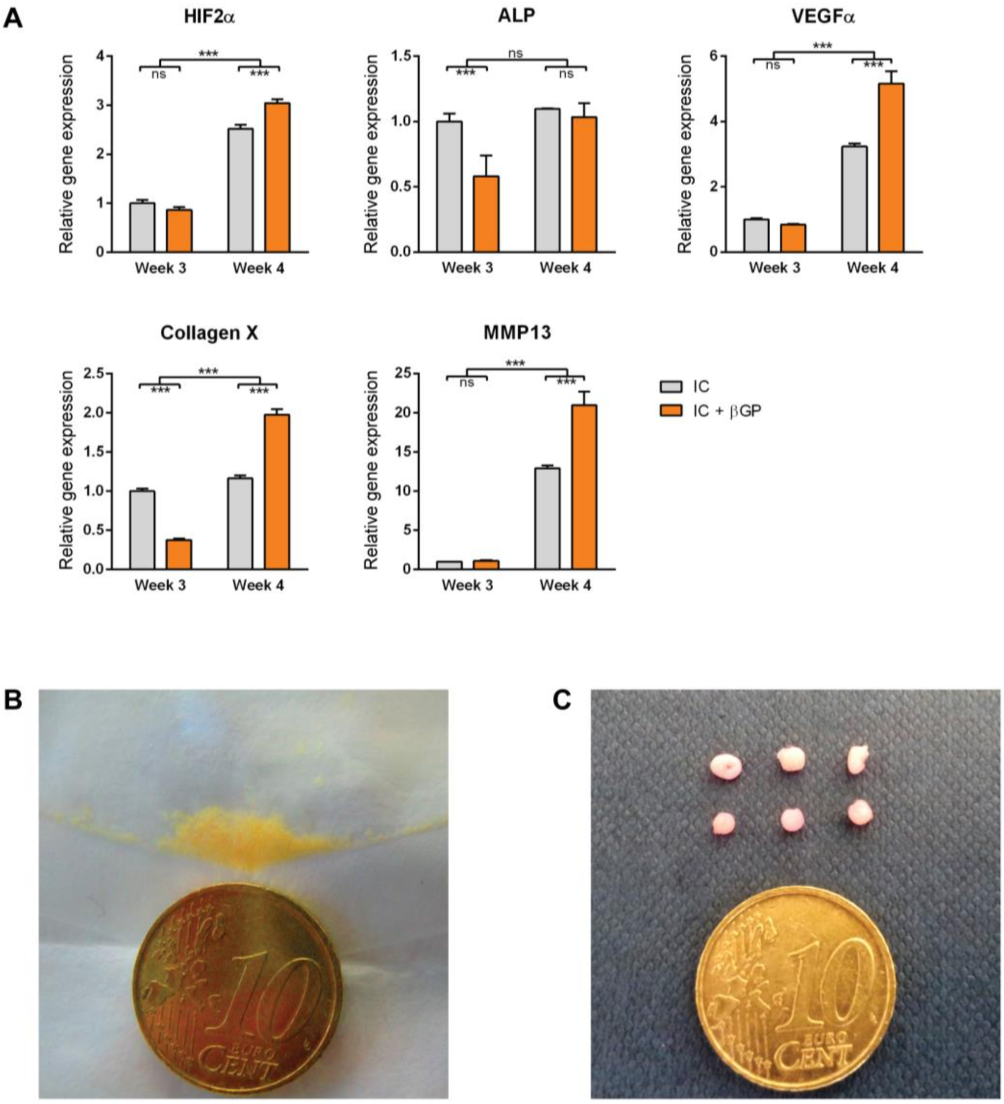

Figure S2. Gene expression analysis of MiTEC cultured in basic medium and mineralization medium (1 week in mineralization medium before time point) at week 3 and 4 **(A)**. Error bars represent standard deviation (n=3). (*) denotes $p<0.05$, (**) denotes $p<0.01$, (***) denotes $p<0.001$, and ns denotes non-significant. MiTEC were cultured in IC medium for 2 weeks, decellularized with SDS and air-dried; the picture showed the amount of MiTEC harvested from one 12-well plate **(B)**. Pellets of $2.5x10^5$ hMSCs mixed with devitalized (top row) and decellularized (bottom row) MiTEC, and cultured in chondrogenic medium for 4 weeks **(C)**.
